# Involvement of Calcium and Calmodulin in NO-Alleviated Salt Stress in Tomato Seedlings

**DOI:** 10.3390/plants11192479

**Published:** 2022-09-22

**Authors:** Nana Qi, Ni Wang, Xuemei Hou, Yihua Li, Weibiao Liao

**Affiliations:** College of Horticulture, Gansu Agricultural University, 1 Yingmen Village, Anning District, Lanzhou 730070, China

**Keywords:** nitric oxide, calcium ion/calmodulin, salt stress, growth, reactive oxygen

## Abstract

Salt stress is an adverse impact on the growth and development of plants, leading to yield losses in crops. It has been suggested that nitric oxide (NO) and calcium ion (Ca^2+^) act as critical signals in regulating plant growth. However, their crosstalk remains unclear under stress condition. In this study, we demonstrate that NO and Ca^2+^ play positive roles in the growth of tomato (*Lycopersicum esculentum*) seedlings under salt stress. Our data show that Ca^2+^ channel inhibitor lanthanum chloride (LaCl_3_), Ca^2+^ chelator ethylene glycol-bis (2-aminoethylether)-N,N,N,N-tetraacetic acid (EGTA), or calmodulin (CaM) antagonist N-(6-aminohexyl)-5-chloro-1-naphthalenesulfona-mide hydrochloride (W-7) significantly reversed the effect of NO-promoted the growth of tomato seedlings under salt stress. We further show that NO and Ca^2+^ significantly decreased reactive oxygen accumulation, increased proline content, and increased the activity of antioxidant enzymes, as well as increased expression of antioxidant enzymes related genes. However, LaCl_3_, EGTA, and W-7 prevented the positive roles of NO. In addition, the activity of downstream target enzymes related to Ca^2+^/CaM was increased by NO under salt stress, while LaCl_3,_ EGTA, and W-7 reversed this enhancement. Taken together, these results demonstrate that Ca^2+^/CaM might be involved in NO-alleviate salt stress.

## 1. Introduction

Salt stress is one of the most important abiotic stresses in plant, which has a series of negative impacts on growth, nutrition, oxidative stress, photosynthesis, respiration, and productivity of many plant species. Salinity could lead to excessive accumulation of sodium (Na^+^) and chloride ion (Cl^−^), thereby damaging the growth and development of plant [1]. In response to salinity, various morphological and developmental changes occur in plant, which implies that plant utilizes an array of biochemical and physiological mechanisms to counteract the harmful effects of salt stress [2]. Moreover, the water and mineral absorption would reduce as salinity increases, which would lead to several consequences to plants, such as nutrient deficiency, decreased chlorophyll content, and decreased function of pigment–protein complex [3]. Additionally, salt stress would increase the risk of oxidative damage in plants that imposes the generation of reactive oxygen species (ROS) [4]. Therefore, salt stress may lead to different degrees of inhibition in plant growth [5]. Consequently, a profound exploration of method to alleviate salt stress is essential for plant growth.

Nitric oxide (NO), a small ubiquitous gaseous signaling molecule, mediates many specific developmental processes in plants. In recent years, NO functions as an influential plant growth regulator and has been widely studied, including seed germination [6], adventitious and lateral root formation [7,8]. Further results confirm that NO is involved in diverse stresses, including drought and osmotic stress, heat, heavy metal, salt and biotic stresses [9,10,11]. Increasing evidence reveals that endogenous NO plays an important role in the signaling network to induce tolerance against salinity in plants. It has also been suggested that exogenous NO application could promote the maintenance of cellular redox homeostasis through increasing antioxidant enzyme activity, and consequently mitigate the oxidative damage produced by ROS in plants under salinity [12]. It was further reported that exogenous NO could increase the activity of antioxidant enzymes in plant, such as superoxide dismutase (SOD), ascorbic acid peroxidase (APX), and catalase (CAT) [13], which were correlated with enhanced tolerance to abiotic stresses. Therefore, the roles of NO in plants are currently a hot topic and require to be urgently studied.

Calcium ion (Ca^2+^), an essential cytoplasmic second messenger, regulates many different responses of plants to environmental signals. Increasing evidence reveals that Ca^2+^ plays an important role in plants response to biotic and abiotic stresses, such as light, oxidative stress, wind, wounding, phytohormones, and pathogens [14]. Ca^2+^ also participates in the growth and development of many plants, including seed germination, root and pollen tube growth [15,16], plant senescence and fruit ripening preservation [17]. Furthermore, several studies have reported that exogenous Ca^2+^ would improve the tolerance to drought stress in different plants such as *Arabidopsis thaliana* [18] and maize [19]. Ca^2+^ signals usually are decoded by several Ca^2+^ sensors or Ca^2+^ binding proteins when plants respond to various stresses. Generally speaking, there are three sub-families of Ca^2+^ sensors and transducers in plants, including calmodulins (CaMs), calcineurin B-like proteins, and calcium-dependent protein kinase (CDPK) [20]. Calmodulin (CaM), a multifunctional Ca^2+^ receptor protein, participates in signaling pathways that regulate many crucial processes [21]. Generally, CaM has no enzymatic activity of its own, but the calcium ion/calmodulin (Ca^2+^/CaM) complex through modulating the activities of numerous target proteins regulates a variety of critical processes [21].

It was demonstrated that a complicated crosstalk exists between NO, Ca^2+^, and CaM in plant growth and response to abiotic stresses [22]. Recently, increasing number of studies dedicated the interplay of NO and Ca^2+^ in plant growth and response to abiotic stress. For example, Lanteri et al. [23] reported that Ca^2+^ and CDPK were involved in NO-induced adventitious root formation in cucumber explants. In addition, adventitious root formation could be induced by exogenous NO through increasing endogenous Ca^2+^ and CaM levels under stress-free condition in marigold [24]. Furthermore, a critical role of Ca^2+^/CaM in NO-induced adventitious root formation was also investigated in cucumber under osmotic stress [25]. However, the interplay between NO and Ca^2+^/CaM remain unclear. So, the aim of this study is to explore the potential role of NO and Ca^2+^/CaM and their interaction in tomato seedlings under salt stress.

## 2. Material and Methods

### 2.1. Plant Materials and Growth Conditions

Tomato seedlings (*Lycopersicum esculentum* L. ‘Micro-Tom’) were used as plant materials in this study, which were cultivated using Hoagland nutrient solution through hydroponic culture. The healthy seeds were surface sterilized with 1% of sodium hypochlorite for 10 min and thoroughly washed with distilled water, and then sown at the nursery trays. After two weeks of germination, they were cultivated in 1/2 Hogland solution for 7 days. Then, they were transferred to whole Hogland solution cultivation for another 21 days. The tomato seedlings were grown under a cycle of temperature 25 ± 2 °C/20 ± 2 °C (day/night), relative humidity 70%, and photoperiod 14/10 h (light/dark).

### 2.2. Treatments

After cultivation in Hoagland solution for 28 days, the uniform size and consistent growth seedlings were chosen for different treatments for 7 days. In order to choose the optimum concentration of CaCl_2_, sodium chloride (NaCl 150 mM) and different concentrations of calcium chloride (CaCl_2_, 50 µM, 100 µM, 150 µM, 200 µM, 250 µM, and 500 µM) were added to Hogland solution. Then, the following chemicals were added to Hogland solution, respectively: 150 µM CaCl_2_, NO donor nitrosoglutathione (GSNO, 10 mM), GSNO+CaCl_2__,_ GSNO together with 1000 µM lanthanum chloride (LaCl_3_, Solarbio), 500 µM ethylene glycol-bis (2-aminoethylether)-N,N,N,N-tetraacetic acid (EGTA, sigma), 200 µM N-(6-aminohexyl)-5-chloro-1-naphthalenesulfona-mide hydrochloride (W-7, Santa Cruz), 10 µM 2-(4-carboxy-2-phenyl)-4,4,5,5-tetramethylimidazoline-1-oxyl-3-oxide (cPTIO, Sigma). The seedlings treated with the Hogland solution adding no extra compounds were served as the control. All the solutions were prepared with distilled water. Each treatment contained three replications. The samples were collected after treatment and frozen at −80 °C for the following experiment. The concentrations of these chemicals were selected based on the results of a preliminary experiment conducted in our laboratory [11,25].

### 2.3. Measurement of Morphological Indexes

The morphological phenotype of seedlings, such as plant height, stem diameter, leaf area, and total root length, were measured after treatment. Plant height and stem diameter were measured by a vernier caliper of straight-line. Leaf area was detected by a leaf area scanner (YMJ-C, Zhejiang Topp Co., Ltd, Hangzhou, China) for leaves of the whole single plant. After removing the aboveground part of the treated seedlings, the root images were scanned with a root scanner (STD4800, Canada), then the root analysis software Win RHIZO 5.0 (Regent Instruments, Inc. Quebec City, Canada) was used to determine the total root length of each plant. Mean values of plant height, leaf area, and total root length in each treatment were calculated by five seedlings in each replication.

### 2.4. Determination of H_2_O_2_ Content, MDA Content and Proline Content

Hydrogen peroxide (H_2_O_2_) content was determined according to Bellincampi et al. [26] with slight modifications. Fresh leaf tissues (500 mg) were homogenized with 5.0 mL of trichloroacetic acid (TCA) (0.1%, *w*/*v*) and then centrifuged at 12,000× *g* for 15 min at 4 °C. Sample extract (0.5 mL) was added to 0.5 mL 10 mM potassium phosphate buffer (pH 7.0) and 1 mL of 1mM potassium iodide. When entirely mixed, it was kept at 28 °C constant temperature for 60 min. The absorbance of the solution was measured at 390 nm. The malondialdehyde (MDA) content was measured as described by Ma et al. [27]. A total of 500 mg of plant leaves were collected and homogenized in 5 mL 100 g·L^−1^ TCA and then transferred into 10 mL centrifuge tube. The mortar was washed using 5 mL TCA and merged in 10 mL centrifuge tube before. Then, the extracts were centrifuged at 4000× *g* for 10 min at 4 °C. The supernatant liquor (2 mL) was added to 2 mL of 6 g·L^−1^ 2-thiobarbituric acid. The mixture was incubated in a boiling water bath for 15 min and then centrifuged for 15 min at 4000× *g* after quickly cooling on ice. The absorbance of the supernatant was measured at 450 nm, 532 nm, and 600 nm. MDA content (nmol·g^−1^) = 6.45 ∗ (OD 532 − OD 600) − 0.56 ∗ OD 450. The proline content was determined according to Myara et al. [28]. Fresh tomato leaves about 500 mg were weighted and ground finely with 5 mL 3% sulfosalicylic acid. After extracting in boiling water bath for 10 min, the homogenate was centrifuged at 3000× *g* for 10 min after cooling. Then, 2 mL glacial acetic acid, 4 mL acid ninhydrin reagent solution and 2 mL 3% (*w*/*v*) sulfosalicylic acid were added to 2 mL of extracting solution, respectively. The mixture was incubated in boiling water bath for 60 min. After cooling the reaction mixture to room temperature, 4 mL toluene was added and allowed to sit until extraction. The red-colored upper phase, which consisted of proline dissolved in toluene, was taken and placed in a spectrophotometer. The absorbance was read at 520 nm. The proline concentration was determined in mg/g fresh leaf weight according to the standard curve.

### 2.5. Assay of Antioxidant Enzymes Activities

Enzyme extracts were prepared by homogenizing on ice using different extraction buffers. About 500 mg leaf samples were homogenized with phosphate buffer (0.05 M, pH 7.8) containing 5 M EDTA-Na_2_, 2 M sodium ascorbate (AsA), and 2% insoluble polyvinylpyrrolidone in a chilled pestle and mortar. The homogenate was centrifuged at 16,000× *g* for 30 min at 4 °C and the resulted supernatant solution was used for determination of superoxide dismutase (SOD), catalase (CAT), and ascorbic acid peroxidase (APX) activity. SOD activity was assayed by the photochemical method described by Nakano and Asada [29]. One unit SOD activity was defined as the amount of enzyme caused 50% inhibition of the rate of nitro blue tetrazolium chloride. CAT activity was determined according to the method of by Nakano and Asada [29] by monitoring the disappearance of H_2_O_2_ by recording the decrease in absorbance at 240 nm. CAT activity was expressed as ∆D240 per minute per milligram of protein. The activity of APX was assayed following the previously described method [30]. The assay mixture consisted of 0.25 mM AsA, 1 M H_2_O_2_ and enzyme extract. APX activity was expressed as ∆OD 290 per minute per milligram of protein. Glutathione reductase (GR) and monodehydroasorbate reductase (MDHAR) activity was measured using an enzyme extraction kit (Suzhou Keming Biotechnology Co., Ltd., Suzhou, China). Enzyme extracts were prepared by homogenizing on ice according to the instruction. About 100 mg leaf sample was ground with 1 mL reagent 1 for ice homogenization. After 4 °C-centrifugation at 8000× *g* for 15 min, the supernatant was used for the subsequent enzyme activity determination using a SHIMADZU UV-1800 spectrophotometer (Shimadzu, Japan). GR and MDHAR activity was calculated using the rate of decrease in the absorbance of NADPH at A340.

### 2.6. Assay for GC, PDE, Ca^2+^-ATPase, PLD, and NADK Activity

The activity of guanylate cyclase (GC), phosphodilipase (PDE), Ca^2+^-ATPase, phospholipase D (PLD), and NAD kinase (NADK) was detected by using ELISA Kits (Yuanmu Biological Technology Co., Ltd., Shanghai, China) according to the manufacturer’s instructions. About 500 mg tomato leaves were ground with 5 mL PBS (pH 7.4) on ice, then rinsed in the mortar with another 4 mL PBS and all the solution was collected into a test tube. The sample was centrifuged at 4000× *g* for 15 min at 4 °C, and the supernatant was collected as crude enzyme solution. In brief, 10 µL of the crude enzyme solution was added into the plate well with 40 µL GC, PDE, Ca^2+^-ATPase, PLD or NADK sample diluent, then reacted at 37 °C for 30 min. Then, the mixture was incubated with 50 μL HRP-conjugate reagent at 37 °C for 30 min. The complex was colored with 50 μL chromogen solution A and 50 μL chromogen solution B at 37 °C for 15 min in the dark. Stop solution 50 μL was added to stop the above reaction. Next, the absorbance was detected at 450 nm using a microplate reader (CMax Plus, Migu Molecular Instruments Co., Ltd., Shanghai, China) within 15 min. The enzyme activity in the sample was determined by comparing the OD 450 of the sample to the standard curve.

### 2.7. Quantitative Real-Time PCR Assays

Quantitative real-time PCR (qRT-PCR) was conducted to assess the transcription levels of different genes. Total RNA was extracted from the homogenized powder of tomato leaves using TRIzol reagent (Invitrogen). The cDNA was synthesized with an Evo M-MLV RT Premix for qPCR Kit (Accurate Biotechnology Co., Ltd., Hunan, China) following the manufacturer’s recommendations. The qRT-PCR experiments were performed on a Light Cycler^®^ 96 Real-Time PCR System (Roche, Switzerland), according to the manufacturer’s instructions in SYBR Green Premix Pro Taq HS qPCR Kit (Accurate Biotechnology). All the qRT-PCR analyses were performed with at least three biological replicates. The expression level of *Sl*ACTIN was used as an internal control to calculate the relative expression. The primers used in the qRT-PCR analyses are listed in Supplementary Table S1. The relative transcript expression level of genes was quantified using 2^−ΔΔCt^ method.

### 2.8. Statistical Analysis

The results are reported as means ± standard error (SE) from at least three time experiments. The analysis of data was performed using SPSS Statistics 22.0 software (SPSS, Inc., Chicago, IL, USA). The significant differences were performed using Tukey’s multiple comparison test (*p* < 0.05).

## 3. Results

### 3.1. Effects of Different Concentrations of CaCl_2_ on Growth of Tomato Seedlings under Salt Stress

**Figure 1:**
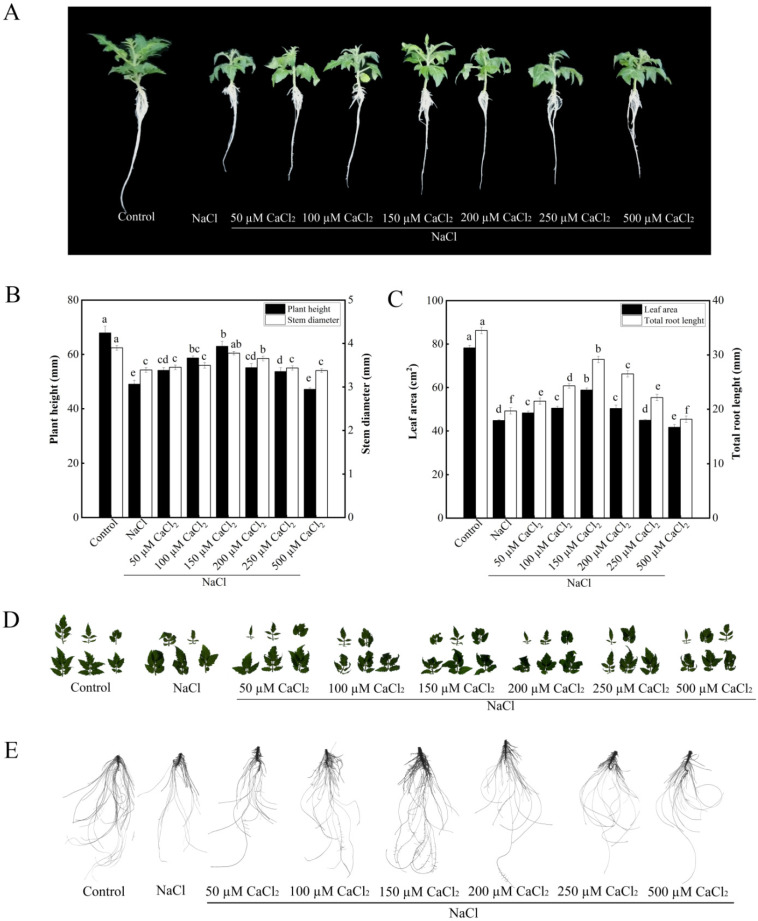
Effects of various concentrations of CaCl_2_ on the growth of tomato seedlings under salt stress. (**A**): Plant phenotype; (**B**): plant height and stem diameter; (**C**): plant leaf area and total root length; (**D**): leaf phenotype; (**E**): root phenotype. The values (means ± SE) are the averages of three independent experiments (*n* = 15). The columns labeled with different small letters indicate significant differences between treatments by Tukey’s multiple comparison test (*p* < 0.05).

Compared with NaCl treatment, 50, 100, 150, and 250 mM CaCl_2_ treatments significantly increased plant height under salt stress (Figure 1A,B). Compared with NaCl treatment, treatments with 150 and 200 mM CaCl_2_ significantly increased stem diameter under salt stress. There was no difference in stem diameter among 50, 100, 250 mM CaCl_2_ and NaCl treatments (Figure 1A,B). Compared with the control, treatment with NaCl significantly decreased leaf area in tomato seedlings. Under salt stress, treatments with 50, 100, 150, 200, and 250 µM CaCl_2_ increased leaf area of tomato seedlings than NaCl treatment. Among them, the leaf area was significantly increased by 31.4% in 150 µM CaCl_2_ treatment under salt stress compared with NaCl treatment. While 500 µM CaCl_2_ treatment decreased leaf area under salt stress more than NaCl treatment alone (Figure 1C,D). Under the NaCl stress, all concentrations of CaCl_2_ significantly increased total root length of tomato seedlings expect the concentration of 500 µM CaCl_2_. The total root length in 150 µM CaCl_2_ treatment was significantly higher than that in 200 µM CaCl_2_ treatment. Treatment with 200 µM CaCl_2_ resulted in higher root length than treatments with 50, 100, and 250 µM CaCl_2_. Thus, 150 µM CaCl_2_ treatment obtained the highest total root length (Figure 1C,E). Thus, 150 µM CaCl_2_ had the maximum biological effect on the growth of tomato seedlings under salt stress. Therefore, 150 µM CaCl_2_ was used for the following experiments.

### 3.2. Effects of Exogenous NO, Ca^2+^ Chelators, Ca^2+^ Channel Inhibitor and CaM Antagonists on Growth of Tomato Seedlings under Salt Stress

**Figure 2:**
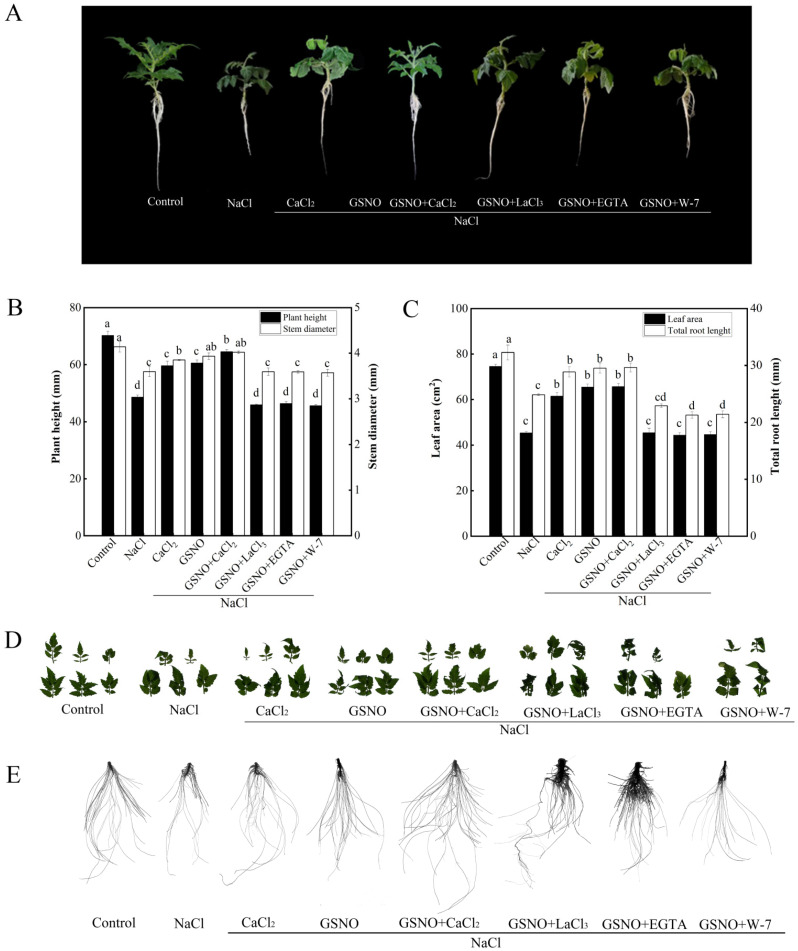
Effects of exogenous NO, Ca^2+^ chelators, Ca^2+^ channel inhibitor and CaM antagonists on growth of tomato seedlings under salt stress. (**A**): Plant phenotype; (**B**): plant height and stem diameter; (**C**): plant leaf area and total root length; (**D**): leaf phenotype; (**E**): root phenotype. The values (means ± SE) are the averages of three independent experiments (*n* = 15). The columns labeled with different small letters indicate significant differences between treatments by Tukey’s multiple comparison test (*p* < 0.05).

In order to further analyze whether Ca^2+^/CaM is involved in NO-promoted the growth of tomato seedlings under salt stress, the effects of Ca^2+^ channel inhibitor (LaCl_3_), Ca^2+^ chelator (EGTA), and CaM antagonist (W-7) co-treated with GSNO were studied. As shown in Figure 2, NaCl treatment significantly decreased plant height, stem diameter, total root length, and leaf area compared with the control. Under salt stress, CaCl_2_, GSNO, and GSNO+CaCl_2_ treatment obtained higher plant height, stem diameter, total root length, and leaf area value than NaCl treatment alone. However, when LaCl_3_, EGTA, or W-7 were added to GSNO containing solution under salt stress, it reversed the GSNO-induced positive effect on tomato seedlings. These results indicated that NO-promoted the growth of tomato seedlings under salt stress relative might be dependent on Ca^2+^/CaM.

### 3.3. Effects of Exogenous NO and Ca^2+^/CaM on the Content of H_2_O_2,_ MDA and Proline in Tomato Seedlings under Salt Stress

**Figure 3:**
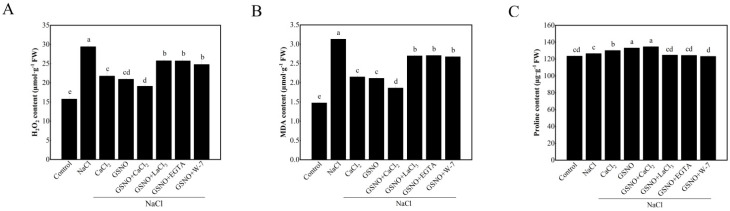
The content of H_2_O_2_, MDA, and proline in tomato seedlings under salt stress. (**A**): H_2_O_2_ content; (**B**): MDA content; (**C**): proline content. The values (means ± SE) are the averages of three independent experiments (*n* = 15). The columns labeled with different small letters indicate significant differences between treatments by Tukey’s multiple comparison test (*p* < 0.05).

As shown in Figure 3A, NaCl treatment significantly increased H_2_O_2_ content in tomato seedlings as compared to the control. Under salt stress, the treatment with CaCl_2_ and GSNO showed lower H_2_O_2_ content compared with NaCl treatment alone. In addition, the co-treatment with GSNO + CaCl_2_ displayed lower H_2_O_2_ content when compared with their alone treatment. Conversely, under salt stress, the content of H_2_O_2_ was higher in GSNO + LaCl_3_, GSNO + EGTA or GSNO + W-7 treatment than in GSNO treatment. NaCl treatment significantly increased MDA content (about 111.9%) compared to the control. Under salt stress, CaCl_2_, GSNO, and GSNO + CaCl_2_ treatments significantly decreased MDA content compared to NaCl treatment. After adding LaCl_3_, EGTA, and W-7 to GSNO, MDA content was significantly increased compared with GSNO treatment under salt stress (Figure 3B). Proline content was higher in NaCl treatment than in the control. CaCl_2_ and GSNO significantly increased the proline content under salt stress. However, LaCl_3_, EGTA, or W-7 reversed the positive roles of GSNO (Figure 3C).

### 3.4. Effects of Exogenous NO and Ca^2+^/CaM on Antioxidant Enzyme Activity of Tomato Seedlings under Salt Stress

**Figure 4:**
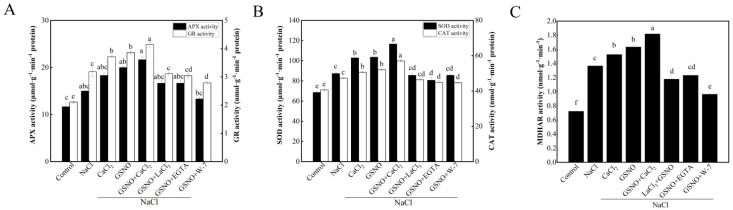
Effects of exogenous NO and Ca^2+^/CaM on antioxidant enzyme activity of tomato seedlings under salt stress. (**A**): APX and GR activities; (**B**): SOD and CAT activities; (**C**): MDHAR activity. The values (means ± SE) are the averages of three independent experiments (*n* = 15). The columns labeled with different small letters indicate significant differences between treatments by Tukey’s multiple comparison test (*p* < 0.05).

To further assess whether alleviation of salt stress is related to NO- or Ca^2+^/CaM -induced antioxidant defense, the activity of antioxidant enzymes was measured in tomato seedlings under salt stress. Compared with the control, the activities of two enzymes of the ascorbate-glutathione pathway, GR and APX, were increased under the NaCl treatment (Figure 4). The activity of APX and GR was increased by CaCl_2_ and GSNO treatment under salt stress (Figure 4A). Compared with GSNO treatment, GSNO+LaCl_3_, GSNO+EGTA, and GSNO+W-7 treatments reduced the activity of APX and GR under salt stress (Figure 4A). As shown in Figure 4B, NaCl treatment led to a significant increase in CAT and SOD activities compared with the control. CaCl_2_ and GSNO increased CAT and SOD activity under salt stress; however, there was a significant decrease in CAT and SOD activity in GSNO+LaCl_3_, GSNO+EGTA, or W-7 treatment. Compared with the control, MDHAR activity was significantly increased in all other treatments (Figure 4C). Compared with NaCl treatment, CaCl_2_, GSNO, and GSNO+CaCl_2_ treatments significantly increased MDHAR activity under salt stress. GSNO+LaCl_3_, GSNO+EGTA, and GSNO+W-7 significantly decreased MDHAR activity compared with GSNO treatment.

### 3.5. Effects of NO on Downstream Target Enzymes Related by Ca^2+^/CaM in Tomato Seedlings under Salt Stress

**Figure 5:**
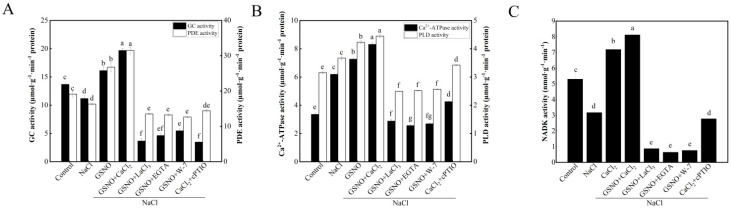
Effects of NO on downstream target enzymes related by Ca^2+^/CaM in tomato seedlings under salt stress. (**A**): GC and PDE activities; (**B**): Ca^2+^-ATPase and PLD activities; (**C**): NADK activity. The values (means ± SE) are the averages of three independent experiments (*n* = 15). The columns labeled with different small letters indicate significant differences between treatments by Tukey’s multiple comparison test (*p* < 0.05).

In comparison with the control, NaCl treatment significantly decreased GC and PDE activity (Figure 5A). When compared with NaCl treatment, CaCl_2_ treatment obviously increased GC and PDE activity by 44.06% and 64.01%, respectively. However, GSNO + LaCl_3_, GSNO + EGTA, or GSNO + W-7 partly reversed the promotion of GSNO. Under salt stress, the activity of GC and PDE in CaCl_2_ + cPTIO treatment was significantly lower than that in CaCl_2_ treatment (Figure 5A). Compared with the control, NaCl treatment significantly increased Ca^2+^-ATPase and PLD activity (Figure 5B). Compared with NaCl treatment, NaCl + CaCl_2_ treatment significantly increased Ca^2+^-ATPase and PLD activity. However, when LaCl_3_, EGTA, or W-7 was administered to GSNO-treated seedlings, it resulted in a significant reduction of Ca^2+^-ATPase and PLD activity under salt stress. In addition, cPTIO significantly decreased Ca^2+^-ATPase and PLD activity which was induced by CaCl_2_ under salt stress. Figure 5C shows an obvious decrease in NADK activity in NaCl treatment, which was 66.83% of the control. Treatment with CaCl_2_ plus NaCl significantly increased NADK activity, which was more than twice than that of NaCl treatment alone. In addition, the NADK activity in seedlings treated with GSNO + LaCl_3_, GSNO + EGTA, or GSNO + W-7 was significantly lower than that in seedlings treated with NaCl. Moreover, CaCl_2_ + cPTIO treatment was able to cause reduction of NADK activity compared with the NaCl treatment.

### 3.6. Effects of Exogenous NO and Ca^2+^/CaM on Antioxidant Synthesis-Related Gene Expression in Tomato Seedlings under Salt Stress

**Figure 6:**
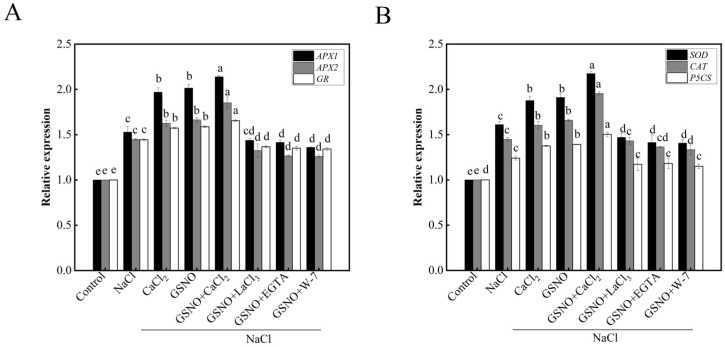
Effects of exogenous NO and Ca^2+^/CaM on antioxidant synthesis-related genes in tomato seedlings under salt stress. (**A**): Relative expression of *APX1*, *APX2*, and *GR*; (**B**): relative expression of *SOD*, *CAT* and *P5CS*. The values (means ± SE) are the averages of three independent experiments (*n* = 15). The columns labeled with different small letters indicate significant differences between treatments by Tukey’s multiple comparison test (*p* < 0.05).

As shown in Figure 6A, the expressions of *APX1*, *APX2,* and *GR*, which encoded APX and GR, respectively, were significantly higher in NaCl treatment than in the control. *APX1*, *APX2,* and *GR* expression levels in NaCl + CaCl_2_ and NaCl + GSNO treatments were higher than that in NaCl treatment. However, the co-treatment with GSNO and LaCl_3_, EGTA, or W-7 obviously reduced the roles of GSNO. Salt stress up-regulated the expressions of *SOD*, *CAT,* and *P5CS* (Figure 6B). While CaCl_2_ and GSNO significantly increased the expressions of *SOD*, *CAT,* and *P5CS* under salt stress. However, when LaCl_3_, EGTA, and W-7 were added, the expressions of *SOD*, *CAT,* and *P5CS* increased by GSNO were decreased under salt stress.

## 4. Discussion

Salt stress is one of the most common abiotic stresses, which has detrimental roles in crop quality and productivity. Therefore, it is imperative to alleviate salt stress in agricultural production. The results clearly showed that salt stress have an adverse effect on the growth of tomato seedlings (Figure 1 and Figure 2). However, GSNO or CaCl_2_ increased plant height, stem diameter, total root length, and leaf area of tomato seedlings under salt stress (Figure 1 and Figure 2). In addition, previous studies have indicated that NO and Ca^2+^, as signaling molecules, might regulate the growth and physiological processes and the response to abiotic stress in plants [25,31]. Similarly, recent studies have shown that NO could promote the growth of tomato seedlings under salt stress [11]. Exogenous Ca^2+^ was also found to promote the adaptive of *Gleditsia sinensis* Lam. under salt stress [32]. Meanwhile, GSNO+CaCl_2_ treatment showed better growth than CaCl_2_ or GSNO treatment alone in tomato seedlings under salt stress (Figure 2). These results indicated that treatment with suitable concentration of NO or Ca^2+^ significantly alleviated the growth inhibition from salt stress in tomato seedlings, and NO and Ca^2+^ have synergistic effects.

Recently, studies have shown an increased interest in the roles and crosstalk between NO and Ca^2+^ response to abiotic stresses in plant. The functional connection between NO and Ca^2+^ in response to salt stress has been reported [33]. Ca^2+^ might mediate NO-induced formation of adventitious root in cucumber under osmotic stress [25]. In this study, when LaCl_3,_ EGTA, or W-7 was applied, the positive effects of NO on the growth of tomato seedlings under salt stress were diminished (Figure 2). These results revealed that Ca^2+^/CaM are essential for NO-alleviated salt stress in tomato seedlings. Similar results were obtained by Niu et al. [25] who reported that LaCl_3,_ EGTA, or W-7 treatment significantly retarded NO-induced the formation of adventitious root in cucumber under osmotic stress. Therefore, it may be hypothesized that Ca^2+^/CaM may be involved in NO-enhanced salt tolerance.

Salt stress might increase the production of ROS which always disrupts the growth and development of plants [34]. This was evident by the increase of H_2_O_2_ and MDA content (Figure 3A,B). These results seem to be consistent with previous study which reported that the content of H_2_O_2_ or MDA was reduced in soybean seedlings under salt stress [35]. In the present study, we observed that the addition of GSNO or CaCl_2_ significantly reduced the production of H_2_O_2_ and MDA under salt stress (Figure 3A,B). A previous study in cucumber showed that H_2_O_2_ content was significantly reduced by SNP or CaCl_2_ treatment during the formation of adventitious root under osmotic stress [25]. Moreover, the data presented here showed that the application of LaCl_3_, EGTA, and W-7 obtained a higher level of H_2_O_2_ and MDA content compared with GSNO or CaCl_2_ treatment. It is possible, therefore, that NO or Ca^2+^ might decrease the H_2_O_2_ and MDA content to protect tomato seedlings under salt stress. Proline, as a component of an antioxidative network, has the ability to protect ROS damage, which participates in mitigating the effect of stress-induced oxidative damage [36]. Earlier study has shown that proline content increased in the leaves of *Brassica* under NaCl treatment [37]. In this work, we found that application of GSNO or CaCl_2_ alone, as well as combination of them all increased the proline content under salt stress (Figure 3C). This result was in line with that of previous study which suggested that presence of GSNO and CaCl_2_ might alleviate NaCl-mediated osmotic stress through enhancing proline accumulation [38]. However, the increase trend of proline content by GSNO was gradually weakened when LaCl_3_, EGTA or W-7 was applied. Thus, these findings suggested that NO or Ca^2+^ might alleviate the adverse effects of salt stress by reducing the accumulation of ROS.

There are several reports demonstrated that NO or Ca^2+^ could regulate the antioxidant system by increasing the activities of antioxidant enzymes to resist damage of abiotic stresses [39]. In the present study, GSNO, CaCl_2_ or GSNO+CaCl_2_ treatment significantly increased the activities of antioxidant enzymes such as APX, SOD, CAT, GR, and DHAR under salt stress (Figure 4), which was consistent with the previous study [40]. In addition, a previous study in tall fescue showed that the effect of GSNO was gradually weakened when the EGTA, LaCl_3_ or PTIO was applied [41]. The result in our study also suggested that LaCl_3_, EGTA or W-7 would decrease GSNO-increased antioxidant enzyme activity under salt stress. These results are in agreement with the hypothesis that Ca^2+^ and NO as signaling molecules enhanced seedlings salt stress resistance by increasing antioxidant enzyme activity [38]; additionally, increasing the expression of genes related to antioxidative enzymes such as *APX1*, *APX2*, *GR*, *SOD*, and *CAT*, as well as proline synthase-associated gene *P5CS* (Figure 6). Therefore, Ca^2+^ and NO alleviated the damage of salt stress by increasing the activity of antioxidant enzymes and the expression of genes related to antioxidative enzymes.

To further investigate the relationships of NO and Ca^2+^/CaM, downstream target enzymes related to Ca^2+^/CaM were determined in our experiment. As a secondary messenger, 3′,5′-cyclic guanosine monophosphate (cGMP) showed a tendency of increase in cytosolic levels with the increase of the salt concentration [42]. Some previous studies have suggested that cGMP might play a pivotal role in plant development processes and responses to both biotic and abiotic stresses [43]. Recent evidence showed that Ca^2+^ and cyclic nucleotides (cAMP/cGMP) cooperate in many plant physiological processes [44]. Interestingly, guanylate cyclases (GCs) are considered to a potential common point of cGMP and NO signaling pathways [45]. Moreover, Ca^2+^ could activate GCs [46]. Ca^2+^/CaM-stimulated PDEs are key enzymes in the superkingdom of eukaryotes. In addition, the activation of PDEs is the result of the attachment of CaM activated by Ca^2+^ [47]. In this study, the result showed that the activity of GC and PDE was decreased in tomato seedlings under salt stress. CaCl_2_ and GSNO+CaCl_2_ increased the activity of GC and PDE under salt stress (Figure 5A). Earlier study has shown that NO could initiate its biological effects mainly through activating GC [48]. Plant Ca^2+^-ATPase, an enzyme actively transport Ca^2+^ in eukaryotic cells, plays a vital role in maintaining cytoplasmic Ca^2+^ homeostasis. Ca^2+^-ATPase might be involved in many biological processes, such as growth, development, hormonal regulation, immunity, and environmental responses [49,50]. In this study, Ca^2+^-ATPase activity was significantly increased by NO, suggesting that Ca^2+^-ATPase might play an important role in NO-alleviated salt stress (Figure 5B). Moreover, Ca^2+^-ATPase was critical for NO-induced freshness preservation in cut lily [51], which showed that NO significantly increased Ca^2+^-ATPase activity. PLD, an important enzyme, is involved in signal transduction, vesicle trafficking, and membrane metabolism [52]. Increasingly, studies have shown the involvement of PLD in plant stress responses or plant defense [53]. In the current study, PLD activity was significantly increased by NO (Figure 5B). In agreement with this finding, NO-induced PLD activation was essential for stomatal closure [54]. NADK, an enzyme identified to be activated by Ca^2+^ -dependent CaM, plays an integral role in plant growth and development [55]. Ca^2+^/CaM complex directly interacts with NADK and activates it in higher plants [56]. The available data in this study showed that CaCl_2_ significantly increased the NADK activity under salt stress (Figure 5C). Therefore, NO could increase the content of Ca^2+^/CaM complex and then activate the activity of NADK. Taken together, these findings suggested that Ca^2+^/CaM might be a downstream molecule involved in NO-alleviate salt stress.

## 5. Conclusions

Altogether, the results of the present study indicated that exogenous NO and CaCl_2_ alleviated the damage of salt stress and promoted the growth of tomato seedlings. In addition, NO and Ca^2+^ alleviated the adverse effects of salt stress mainly by reducing the accumulation of ROS. Moreover, the activity of downstream target enzymes related to Ca^2+^/CaM was increased by NO under salt stress. Our results further demonstrated that Ca^2+^/CaM might be located downstream of NO signaling pathway during that process. However, complicated interactions might exist between NO and Ca^2+^/CaM under stress condition. Therefore, more mechanisms of the crosstalk between NO and Ca^2+^/CaM under various stresses should be further demonstrated.

## Data Availability

The data that support this study will be shared upon reasonable request to the corresponding author.

## Supplementary Materials

The following supporting information can be downloaded at: https://www.mdpi.com/article/10.3390/plants11192479/s1, Table S1: Primers used in quantitative real-time PCR (qRT-PCR).
